# Molecular cytogenetic analysis reveals the existence of two independent neo-XY sex chromosome systems in Anatolian Pamphagidae grasshoppers

**DOI:** 10.1186/s12862-016-0868-9

**Published:** 2017-02-07

**Authors:** Ilyas Yerkinovich Jetybayev, Alexander Gennadievich Bugrov, Mustafa Ünal, Olesya Georgievna Buleu, Nikolay Borisovich Rubtsov

**Affiliations:** 1 Institute of Cytology and Genetics, Russian Academy of Sciences, Siberian Branch, Pr. Lavrentjeva 10, 630090 Novosibirsk, Russia; 2 Institute of Systematics and Ecology of Animals, Russian Academy of Sciences, Siberian Branch, Frunze str. 11, 630091 Novosibirsk, Russia; 3 Novosibirsk State University, Pirogov str., 2, 630090 Novosibirsk, Russia; 4 Fen-Edebiyat Fakültesi, Biyoloji Bölümü, Abant İzzet Baysal Üniversitesi, TR-14030 Bolu, Türkiye

**Keywords:** Pamphagidae grasshoppers, Karyotype, Neo-sex chromosome evolution, The neo-X, The neo-Y, FISH, rDNA, Telomeric repeats

## Abstract

**Background:**

Neo-XY sex chromosome determination is a rare event in short horned grasshoppers, but it appears with unusual frequency in the Pamphagidae family. The neo-Y chromosomes found in several species appear to have undergone heterochromatinization and degradation, but this subject needs to be analyzed in other Pamphagidae species. We perform here karyotyping and molecular cytogenetic analyses in 12 Pamphagidae species from the center of biodiversity of this group in the previously-unstudied Anatolian plateau.

**Results:**

The basal karyotype for the Pamphagidae family, consisting of 18 acrocentric autosomes and an acrocentric X chromosome (2n♂ = 19, X0; 2n♀ = 20, XX), was found only in *G. adaliae*. The karyotype of all other studied species consisted of 16 acrocentric autosomes and a neo-XY sex chromosome system (2n♂♀ = 18, neo-XX♀/neo-XY♂). Two different types of neo-Y chromosomes were found. One of them was typical for three species of the *Glyphotmethis* genus, and showed a neo-Y chromosome being similar in size to the XR arm of the neo-X, with the addition of two small subproximal interstitial C-blocks. The second type of the neo-Y chromosome was smaller and more heterochromatinized than the XR arm, and was typical for all Nocarodeini species studied. The chromosome distribution of C-positive regions and clusters of ribosomal DNA (rDNA) and telomeric repeats yielded additional information on evolution of these neo-XY systems.

**Conclusion:**

Most Pamphagidae species in the Anatolian region were found to have neo-XY sex chromosome systems, belonging to two different evolutionary lineages, marked by independent X-autosome fusion events occurred within the Trinchinae and Pamphaginae subfamilies. The high density of species carrying neo-XY systems in the Anatolian region, and the different evolutionary stage for the two lineages found, one being older than the other, indicates that this region has a long history of neo-XY sex chromosome formation.

## Background

The karyotype structure of grasshoppers is very conservative. For instance, the modal male karyotype of Acrididae grasshoppers consists of 22 acrocentric autosomes and an acrocentric X chromosome. In Pyrgomorphidae and Pamphagidae, the modal karyotype consists of 18 acrocentric autosomes and an acrocentric X chromosome. The sex chromosome system in the vast majority of species is XX♀/XO♂ [1–4]. In the karyotype evolution of some species the X chromosome enters into centric fusion with an autosome, which leads to a neo-XX♀/neo-XY♂ sex chromosome system. In some species, this has resulted in new chromosome races [5–7] while in some other species neo-sex chromosomes have become a species karyoptypic feature (see review:[8]). At present, neo-sex chromosomes have been described in more than 100 species from different taxonomic groups of grasshoppers [1, 2, 8, 9]. However, it is rare for neo-sex chromosomes to characterize a group of closely related species. Furthermore, after chromosome fusion, the neo-sex chromosome usually does not exhibit further evolutionary change. This observation suggests that *de novo* originated neo-sex chromosomes are evolutionary dead ends which do not lead to species divergence [10]. For a long time, neotropical species belonging to the Melanoplinae subfamily of Acrididae grasshoppers were an exception to this rule. In this group, with a basal XX♀/XO♂ sex chromosome system, many species have a neo-XX♀/neo-XY♂ sex chromosome system. Furthermore, in some species, neo-Y chromosome-autosome fusion has resulted in a more advanced neo-X_1_X_1_X_2_X_2_♀/neo-X_1_X_2_Y♂ sex chromosome system [8, 9]. Cytogenetic studies of Pamphagidae grasshoppers in the Palearctic region indicates that this group is another example of sex chromosome evolution. The neo-XX♀/neo-XY♂ sex chromosome system was revealed in 11 of 43 studied species of the Pamphagidae family [11–22]. In one species, a neo-X_1_X_1_X_2_X_2_♀/neo-X_1_X_2_Y♂ sex chromosome system was described [22]. Species with neo-sex chromosomes belonged to different subfamilies, Trinchinae and Pamphaginae. What is more, within Trinchinae, neo-sex chromosomes were observed in 4 species of the *Asiotmethis* genus and in one closely related monotypic *Atrichotmethis* genus, while in Pamphaginae neo-sex chromosomes were observed in all studied species belonging to the Nocarodeini tribe [14, 18, 19, 22]. Neo-Y chromosomes in these groups exhibit different morphology indicating further intensive reorganization after formation, resulting in heterochromatinization and shrinkage of the neo-Y chromosome.

In early comparative studies of Pamphagidae grasshoppers, it was suggested that species with neo-sex chromosomes belong to monophyletic group. And species from Trinchinae subfamily, showing less heterochromatinized neo-Y chromosome, are an initial stage of more heteromochromatinized neo-Y chromosome in Nocarodeini species [19]. This suggestion was made on an assumption of low possibility of such rare event as neo-sex chromosome formation in different clades of one family.

These findings make Pamphagidae grasshoppers an interesting model for studying sex chromosome evolution. However, detailed cytogenetic analysis of grasshoppers’ karyotypes is hindered by lack of chromosome markers. Despite wide range of classical chromosome bandings used in cytogenetics, only C-banding, that reveals regions enriched with C-heterochromatin, can be used in grasshoppers. Molecular cytogenetic methods can overcome these difficulties through mapping molecular markers, such as repetitive or unique sequences on chromosomes. This approach has been successfully applied to different groups of grasshoppers including species of neotropical Melanoplinae subfamily with neo-sex chromosome systems [22–28]. Therefore, application of molecular cytogenetic methods as well as classical banding - is crucial for full understanding of sex chromosome evolution in yet unstudied species of this family.

Analysis of the geographical distribution of Pamphagidae grasshoppers showing neo-sex chromosomes indicated that some species of the Thrinchinae subfamily and all the Nocarodeini tribe (Pamphaginae subfamily) species studied occur in South Eastern Europe, Central Asia and the Caucasian Mountains [14, 18, 19, 22]. The number of species with neo-sex chromosomes was found to be high near the Western Asian region, yet this region remained cytogenetically unstudied. We thus focused our research on Pamphagidae grasshoppers from Western and Central Anatolia, to test the hypothesis that neo-sex chromosome formation may have taken place during the evolution of Pamphagidae grasshoppers in the Western Asian region.

## Methods

Males of four species belonging to the Trinchinae subfamily and 8 species (two of them having two subspecies) from the Pamphaginae subfamily were collected during summer season 2014 in Western and Central Anatolia (Table 1). In addition to males, females of *Oronothrotes furvus* were captured and kept in a cage with moisturized sand for egg-pod laying. Chromosome preparations and C-banding were performed as described earlier [22].

**Table 1:** Location and specimens’ number of Pamphagidae species studied in this work

Subfamily. Tribe	Species	Location	Specimens’ number
Trinchinae	*Glyphotmethis adaliae* (Uvarov, 1928)	37.37.518 N 29.13.948 E	6
	*Glyphotmethis dimorphus dimorphus* (Uvarov, 1934)	38.18.438 N 31.43.676 E	11
	*Glyphotmethis efe* (Ünal, 2007)	39.03.285 N 29.26.741 E	10
	*Glyphotmethis holtzi pulchripes* (Uvarov, 1943)	38.46.688 N 34.51.215 E	14
Pamphaginae, Nocarodeini	*Oronothrotes furvus* (Mishchenko, 1951)	38.21.258 N 28.06.713 E	27
	*Nocaracris sp*.	38.16.672 N 31.19.491 E	7
	*Paranocaracris citripes citripes* (Uvarov, 1949)	37.05.779 N 28.50.972 E	13
	*Paranocaracris citripes idrisi* (Karabağ, 1953)	40.35.385 N 31.17.293 E	9
	*Paranocaracris sureyana* (Ramme, 1951)	39.02.353 N 29.17.074 E	3
	*Paranocarodes fieberi anatoliensis* (Demirsoy, 1973)	37.48.527 N 30.45.472 E	2
	*Paranocarodes fieberi tolunayi* (Karabag, 1949)	40.40.937 N 31.46.489 E	2
	*Paranocarodes straubei* (Fieber, 1853)	39.54.453 N 30.41.477 E	1
	*Paranocarodes turkmen* (Ünal, 2014)	39.54.453 N 30.41.477 E	2
	*Pseudosavalania karabagi* (Demirsoy, 1973)	39.03.285 N 29.26.741 E	15

An rDNA probe was generated through PCR of specific fragments of the 18S rRNA gene with specific primers (Table 2). Primers were designed in the PerlPremier software [29], using 18S rDNA consensus sequence from aligning 45S rDNA sequences of four sequences of different grasshoppers (gb|AY379758.1, gb|KM853211.1, gb|KF855839.1 and gb|JF792554.1) using the Mulalin software, (http://multalin.toulouse.inra.fr/multalin/) [30].

**Table 2:** Primers used for 18S rDNA amplification

Primer	Sequence	Product size
18S–1f	5′-ATGGTTCCTTAGATCGTACCC-3′	741 b.p.
18S–1r	5′-TTGTCAAAGTAAACGTGC-3′	
18S–2f	5′-GCATGGAATAATGGAATAGGAC-3′	667 b.p.
18S–2r	5′-AGAACATCTAAGGGCATCAC-3′	
18S–3f	5′-TGATAGCTCTTTCTTGATTCGG-3′	506 b.p.
18S–3r	5′-AGTTTGGTCATCTTTCCGGT-3′	

Three DNA fragments were amplified separately in 20 μl of reaction mix in 25 cycles of polymerase chain reaction (PCR) (initial denaturation 3 min 95 °C, cycles 1 min−95 °C, 40 s−58 °C, 1 min−72 °C, and final elongation 8 min−72 °C) from 40 ng of genomic DNA of *Chorthippus biguttulus* (*Linnaeus*, 1758). PCR mix contained 1 × Taq polymerase buffer, 2 mM MgCl_2_, 0.2 mM dNTP, 0.5 μM of each primer and 0.03 U/μl Taq DNA polymerase (Medigen, Novosibirsk, Russia). Labeling was performed in 25 additional cycles of PCR. The reaction mix was similar to that described above, but the concentration of dTTP was reduced to 0.15 mM and 0.05 mM Fluorescein-dUTP (Medigen, Novosibirsk, Russia) was added and the concentration of Taq DNA polymerase was increased to 0.06U/μl. The reaction mix from the previous step was diluted 100 fold and 1 μl of it was added to 19 μl of labeling reaction mix as a matrix. For hybridization, an equal amount of labeling reaction mix was mixed together and was used as a DNA probe.

Insect telomeric repeats (TTAGG)_n_ were generated in non-template PCR with 5′-TAACCTAACCTAACCTAACC-3′ and 5′-TTAGGTTAGGTTAGGTTAGG-3′ primers according to standard protocol with modifications [31]. Labeling was performed with Tamra-dUTP (Medigen, Novosibirsk, Russia) in additional cycles of PCR, as described earlier [32].

Fluorescence in situ hybridization (FISH) was carried out as was described earlier [32, 33]. DAPI counterstaining was performed after FISH using Vectashield antifade containing 4′,6-diamidino-2-phenylindole (DAPI) (Vector laboratories, USA) under cover glass which was then sealed with rubber cement.

Microscopic analysis was carried out at the Centre for Microscopy of Biological Subjects (Institute of Cytology and Genetics, Novosibirsk, Russia). Chromosomes were studied with an Axio Imager. M1 (Zeiss) fluorescence microscope equipped with filter sets #49, #46HE, #43HE (ZEISS), ProgRes MF (Meta Sistems) CCD camera. The ISIS5 software package (MetaSystems GmbH, Germany) was used for image capture and analysis.

The nomenclature of chromosomes suggested for Pamphagidae grasshoppers [12] was used for the description of chromosomes and karyotypes. According to this nomenclature, autosomes were numbered in order of decreasing size (1–9) and classified into three size groups: L – large, M – medium and S – small. In the species with neo-sex chromosomes they were named following White [34]. The arms of the submetacentric neo-X chromosome were referred to as XL and XR. The short arm (XL) corresponds to the former acrocentric X chromosome and the long arm (XR) to the translocated acrocentric autosome. The unfused autosome, homologous to the XR arm, remains acrocentric and is the neo-Y chromosome.

## Results

The Pamphagidae grasshoppers from Western and Central Anatolia analyzed in the current study belonged to Trinchinae (four species) and Pamphaginae (Nocarodeini tribe, 8 species, two of them having two subspecies). These species had not hitherto been cytologically analyzed, and we provide here the first information on chromosome number, morphology and structure, using several techniques including Giemsa staining, C-banding and FISH for ribosomal DNA (rDNA) and telomeric repeats.

### Karyotypes of studied species

**Fig. 1:**
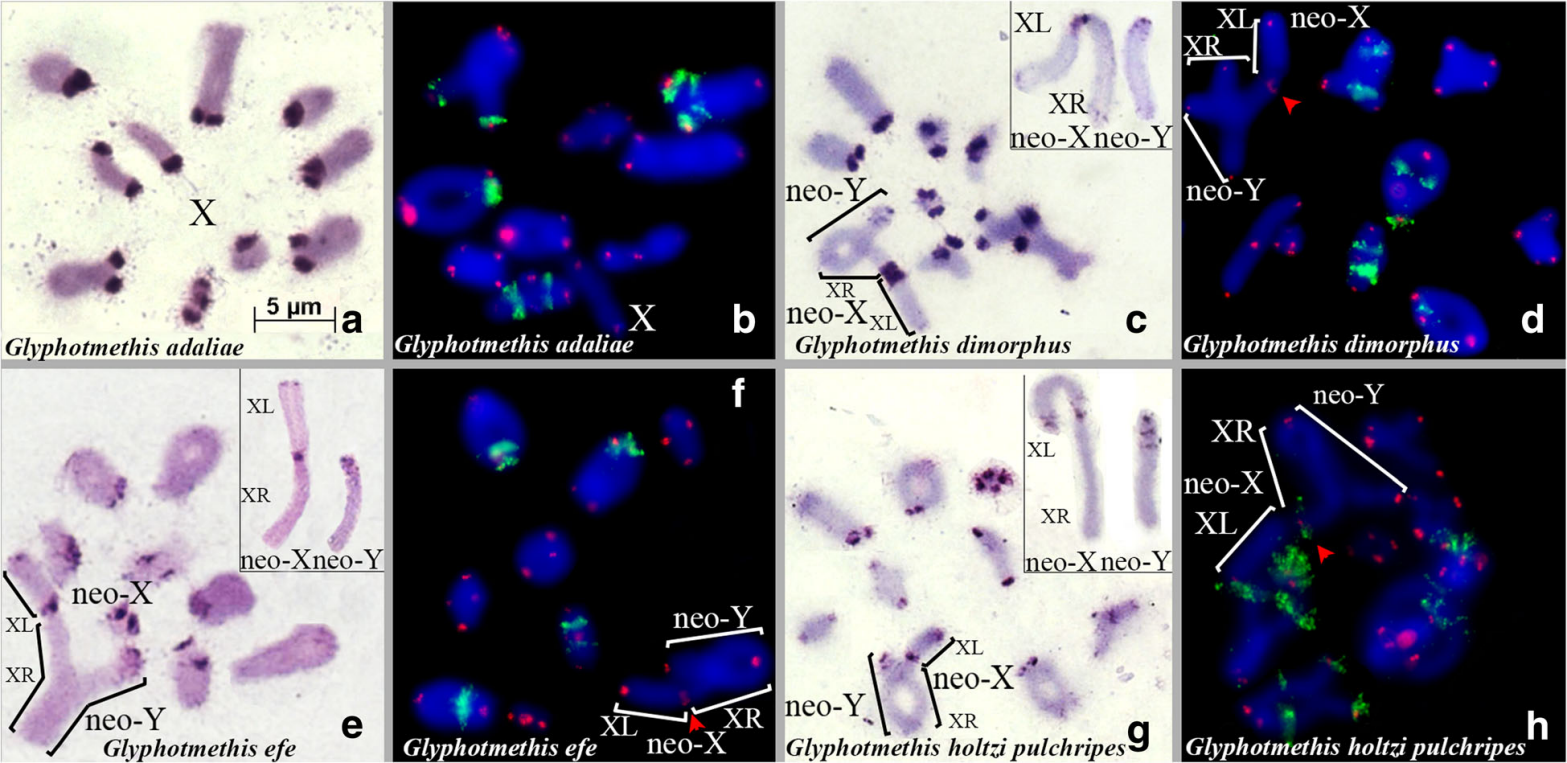
C-banding (**a**,**c**,**e**,**g**) and fluorescence in situ hybridization (FISH) with both rDNA (*green*) and telomeric DNA (*red*) probes (**b**,**d**,**f**,**h**) in diakinesis/metaphase I for the following *Glypotmethis* species from Trinchinae subfamily: *G. adaliae* (**a**,**b**); *G. dimorphus* (**c**,**d**); *G. efe* (**e**,**f**); *G. holtzi pukhripes* (**g**,**h**). *Red* arrowheads indicate ITSs. The inset in the top right corner shows neo-X and neo-Y chromosomes in mitotic metaphase

The Trinchinae species exhibited two types of karyotype. The first type was found only in *Glyphotmethis adaliae* and consisted of 18 acrocentric autosomes (L_1_–L_4_, M_5_–M_8_, S_9_) and a medium sized acrocentric X chromosome. The sex chromosome system was XX♀/X0♂. (Figure 1a, b). The second type of karyotype, found in *Glyphotmethis dimorphus*, *Glyphotmethis efe*, *Glyphotmethis holtzi pulchripes*, consisted of 16 autosomes (L_1_–L_3_, M_4_–M_7_, S_8_), a submetacentric neo-X chromosome and a large acrocentric neo-Y chromosome (Fig. 1 c–h). The sex chromosome system was neo-XX♀/neo-XY♂. The size of this neo-Y is equal to the XR arm of the neo-X chromosome, and during meiosis the neo-Y and XR arm formed a normal bivalent with 1–2 chiasmata.

**Fig. 2:**
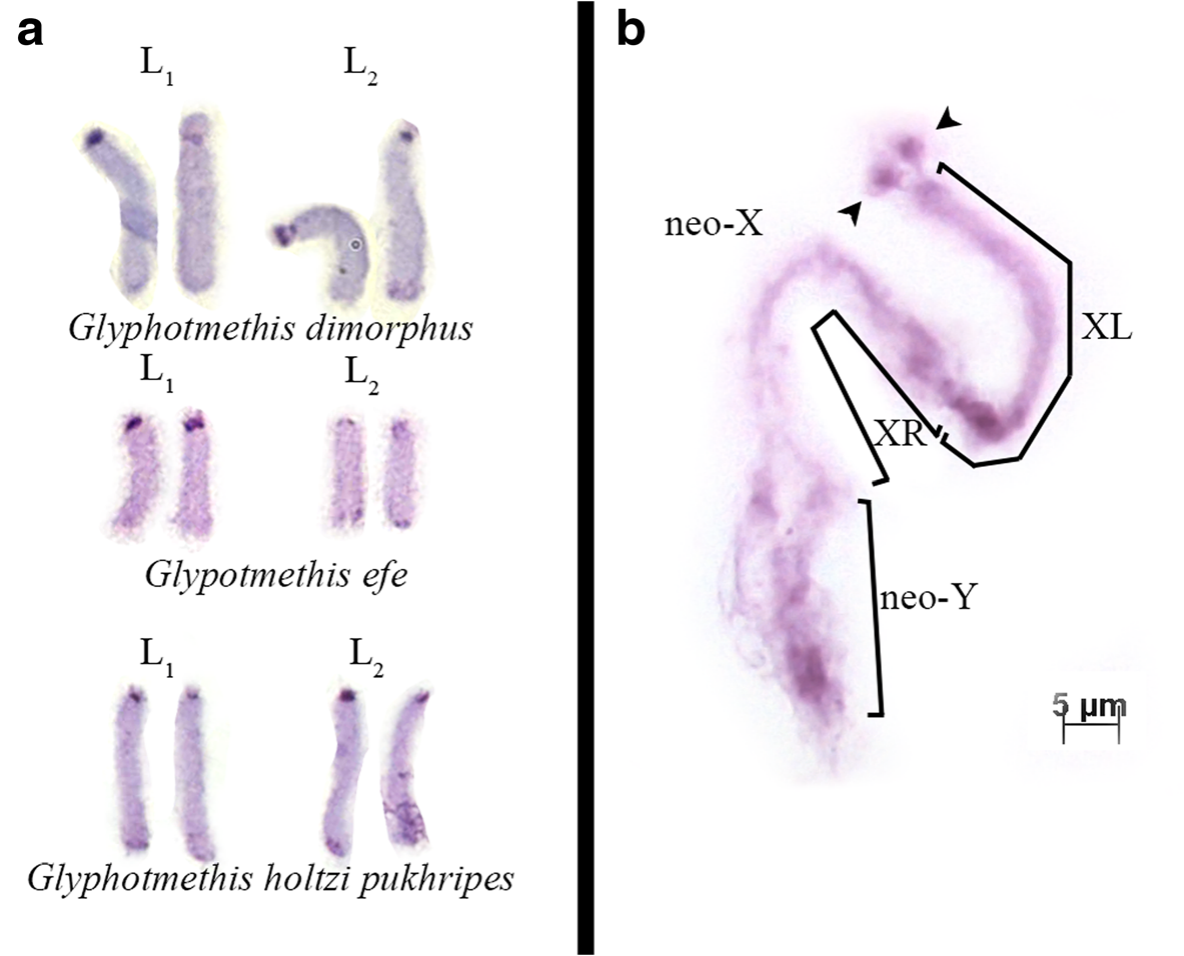
Features of karyotypes of species studied. **a** L_1_ and L_2_ chromosome pairs with polymorphic small second arms in species form *Glyphotmetis* genus; **b** Neo-X-neo-Y bivalent of *Nocaracris sp*. in early diplotene. Arrowheads indicate conjugation of terminal part of XL arm of neo-X chromosomes with two small dot-like B-chromosomes

Large chromosomes in these three Trinchinae species were characterized by small short arms showing variation in size (Fig. 2a). In *G. dimorphus* and *G. efe*, the short arm in the L_1_ chromosome was polymorphic whereas in *G. holtzi pulchripes* all L_1_ chromosomes showed similar-sized short arms. In the L2 chromosome also showed a conspicuous short arm in *G. dimorphus* and *G. holtzi pulchripes*.

**Fig. 3:**
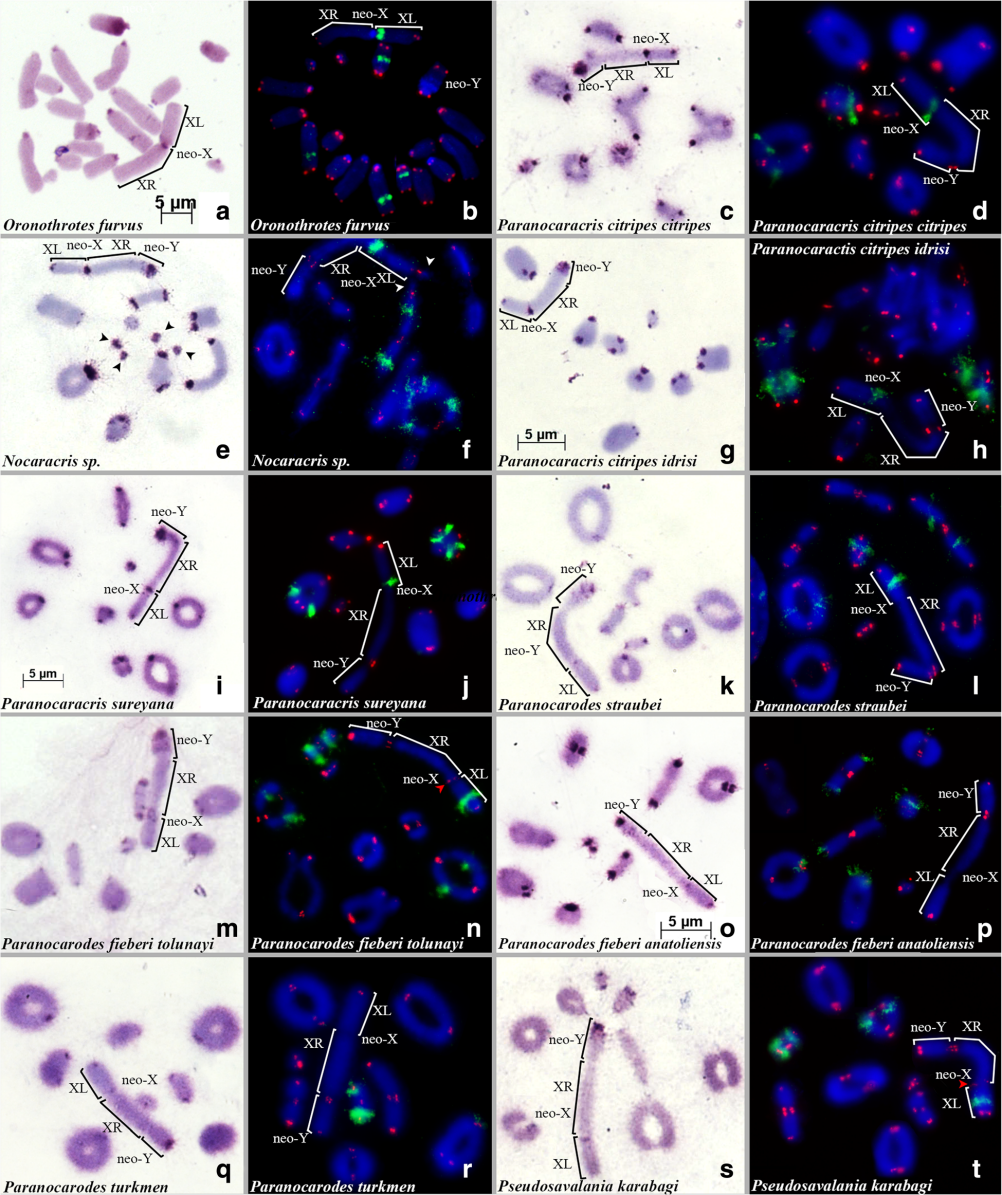
C-banding (**a**,**c**,**e**,**g**,**i**,**k**,**m**,**o**,**q**,**s**) and FISH using both rDNA (*green*) and telomeric (*red*) probes (**b**,**d**,**f**,**h**,**j**,**l**,**n**,**p**,**r**,**t**) of chromosomes in Nocarodeini tribe: *Oronothrotes furvus* (**a**,**b**); *Paranocaracris cytripes cytripes* (**c**,**d**); *Nocaracris sp*. (**e**,**f**) - arrowheads indicate dot-like B-chromosomes; *P. cytripes idrisi* (**g**,**h**); *P. sureyanus* (**i**,**j**); *Paranocarodes fieberi anatoliensis* (**k**,**l**); *P. fieberi tolunai* (**m**,**n**); *P. straubei* (**o**,**p**); *P. turkmen* (**q**,**r**); *Pseudosavalania karabagi* (**s**,**t**). *Red* arrowheads indicate ITSs (**n** and **t**)

The karyotypes of all Pamphaginae species analyzed here consisted of 16 acrocentric autosomes (three chromosome pairs being large acrocentrics (L_1_–L_3_), four pairs being medium acrocentrics (M_4_–M_7_) and one pair being small sized acrocentrics (S_8_)), a submetacentric neo-X chromosome and a medium sized acrocentric neo-Y chromosome. The size of the neo-Y chromosome was smaller than the XR arm and, during meiosis, the sex bivalents formed only one chiasma in the distal region of the neo-Y (Fig. 3). In one of the species studied (*Nocaracris sp*.) 1–4 dot-like B chromosomes were found in 6 out of the 7 specimens analyzed (Fig. 3e, f). In diplotene these chromosomes showed association with terminal part of XL arm (Fig. 2b).

### C-heterochromatin variation

C-banding in the four Trinchinae species showed variation in size and location of C-heterochromatin between species. The C-banding pattern in *G. adaliae*, the only species showing the typical pamphagid karyotype (2n = 18 + X0), showed C-bands localized on pericentromeric regions of all chromosomes, with only an additional C-band on the distant end of the smallest chromosome (Fig. 1a).

The autosomes of *G. dimorphus*, *G. holtzi pulchripes*, and *G. efe* showed C-bands varying in number, size, and location, most being pericentromeric excepting a distal C-band in the L_2_ autosome. Pericentromeric C-bands in the autosomes of *G. dimorphus* were larger than those in *G. holtzi pulchripes*, and *G. efe*. In *G. holtzi pulchripes*, additional distal C-bands were found in the L_1_ and S_8_ chromosomes, and also an interstitial C-band in the L_2_ located close to the distal C-band.

The C-banding pattern in the neo-sex chromosome showed scarce variation in these three species. The size of the pericentromeric C-positive block of the neo-X chromosome was similar to those on the autosomes and was large in *G. dimorphus* and small in *G. efe* and *G. holtzi pulkhripes*. All three species showed a distal C-band on the XL arm, and *G. holtzi pulkhripes* additionally showed polymorphic subproximal and subdistal C-bands. The XR was C-negative excepta small distal C-band only in *G. dimorphus*. The neo-Y chromosome showed a small pericentric C-positive block and two small interstitial C-bands next to the pericentromeric region (Fig. 1c, e, g,). The neo-Y chromosome in *G. dimorphus* showed a small distal C-band Which was not observed in the two other species (Fig. 1c).

**Fig. 4:**
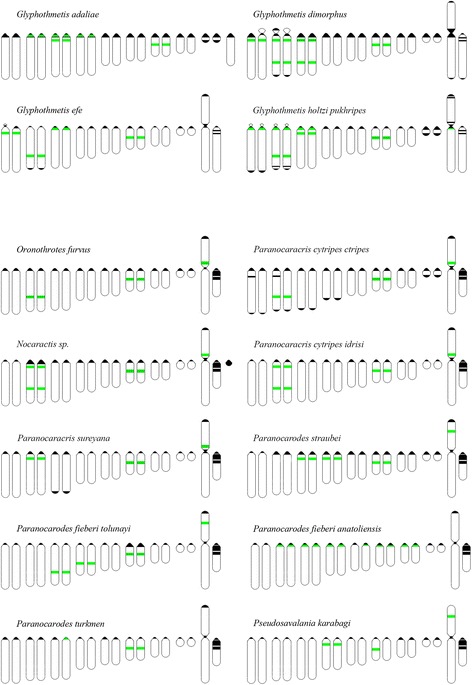
Ideograms of karyotypes of Pamphagidae grasshoppers. *Black* blocks indicates C-bands, *green* blocks indicate rDNA clusters. *Striped* blocks indicate colocalisation of rDNA clusters and C-bands. Block on one chromosome indicates polymorphic sites

In the Pamphaginae species, C-positive bands were mostly located on pericentromeric regions, with some size differences among species. In addition to the pericentromeric C-bands, some interstitial and distal C-bands were observed (Figs. 3, and 4). For instance, *O. furvus*, *P. cytripes cytripes*, *Nocaracris sp*., *P. sureyana*, and *P. fieberi tolunayi* showed interstitial C-bands in the middle of the M_6_ chromosome. Likewise, *O. furvus* showed an additional interstitial C-band on the L_2_ chromosome. In some specimens of *P. cytripes cytripes*, additional interstitial C-bands were observed on proximal regions of the L_1_ and L_2_ chromosomes. Finally, distal C-bands were observed on five chromosome pairs in *P. cytripes cytripes* and on the L_3_ chromosome of *P. sureyana*. The dot-like B chromosomes revealed in *Nocaracris sp*. (Figure 3e, f) were C-positive, indicating their heterochromatic nature.

The neo-X chromosome in Nocarodeini species showed a small or medium sized pericentromeric C-band. In *O. furvus* and *P. sureyana*, a subproximal interstitial C-band was observed in the XL arm. Distal C-bands in the XL arm of the neo-X were identified in 9 species (*O. furvus*, *P. cytripes cytripes*, *Nocaracris sp*., *P. cytripes idrisi*, *P. sureyana*, *P. pahlagonicus*, *P. turkmen*, *P. fieberi tolunayi*, and *P. fieberi anatoliensis*). In all ten species, the neo-Y chromosome showed a large pericentromeric C-band and two or three large subproximal interstitial C-bands located close to each other. In highly condensed chromosomes, these blocks usually merge into one large C-positive region constituting almost half of the neo-Y chromosome (Fig. 3).

### rDNA clusters in chromosomes of Pamphagidae grasshoppers

The number of rDNA clusters in species belonging to the Trinchinae subfamily varied from 4 to 7, with extensive variation in chromosome location (Figs. 1 and 4). Many rDNA clusters were located on pericentromeric and interstitial regions of large autosomes, the M_6_ chromosome and the XL arm, at any location. In most cases, every chromosome carried a single rDNA cluster, but two rDNA clusters were observed in a same chromosome arm in three Trinchinae species (*G. adaliae*, *G. dimorphus* and *G. holtzi pulchripes*) (Fig. 1 b, d, h). Furthermore, in *G. dimorphus* and *G. holtzi pulchripes* we found two pairs of autosomes containing two rDNA clusters in the same chromosome arm, although the precise position of rDNA clusters on these chromosomes differed. While in *G. dimorphus* all rDNA clusters were found on interstitial regions, in *G. adaliae* and *G. holtzi pulchripes* the locations of rDNA clusters were pericentromeric and interstitial.

In species belonging to the Nocarodeini tribe, within the Pamphaginae subfamily, the number of rDNA clusters varied from 2 to 6 (Figs. 3, 4). The level of variation in rDNA cluster location was also high; however, the four *Paranocaracris* species showed higher similarity in rDNA cluster location than the four *Glyphotmethis* species. Some of the conserved locations in *Paranocaracris* species were also observed in other Pamphaginae. For instance, *O. furvus* and *Nocaracris sp*. also showed rDNA clusters on L_2_ and M_6_ chromosomes, as well as on the proximal region of the XL arm (Fig. 3b, d, f, h, j), however the location on L_2_ being variable. In *Nocaracris sp*. and *P. cytripes idrisi*, two rDNA clusters were located on this same chromosome, whereas, in *O. furvus*, *P. sureyana*, and *P. cytripes cytripes*, the only rDNA cluster was either proximal or distal on L_2_ (Fig. 3d, h). In *Paranocarodes* species, we found higher variation than in *Paranocaracris*. For instance, in *P. straubei* and *P. fieberi tolunayi*, L_2_, L_4_ and M_6_ chromosomes, and the XL arm, carried rDNA clusters, although they were located on different chromosome locations (Fig. 3l , n). In *P. fieberi anatoliensis* and *P. turkmen*, the location of rDNA clusters differed significantly compared to other Nocarodeini species (Fig. 3p, r). In *P. turkmen*, only the M_6_ chromosome pair carried an rDNA cluster, whereas the cluster on the pericentromeric region of the L_3_ chromosome was very small and polymorphic. Finally, almost all chromosomes in *P. fieberi anatoliensis* carried pericentromeric rDNA clusters, except L_1_, S_8_, neo-X and neo-Y. In *P. karabgi* rDNA distribution was similar to *P. straubey* and *P. fieberi tolunai*, but no rDNA cluster was observed on L_3_ chromosome (Fig. 3t).

### Telomeric DNA clusters in Pamphagidae grasshoppers

In situ hybridization of the telomeric DNA probe on chromosomes of the species studied here, revealed fluorescent signals on the ends of all chromosomes. However, in five species (three species of *Glyphotmethis* genus (*G. dimorphus*, *G. efe* and *G. holtzi pulchripes*) (red arrows on Fig. 1d, f, h), *P. fieberi tolunayi* and *P. karabagi* (red arrow on Fig. 3n, t)) an additional FISH signal of telomeric repeats was observed on pericentromeric regions of the neo-X chromosome. In addition, *G. holtzi pulchripes* showed interstitial telomeric sequences (ITS) on the subtelomeric region of the XL arm (Fig. 1h).

## Discussion

### The neo-sex chromosome systems in Pamphagidae grasshoppers

The X0/XX sex chromosome system had been reported for 15 Trinchinae and 16 Pamphaginae species previously studied, thus being considered the standard sex chromosome system for this grasshopper family [11–13, 15, 17, 20–22, 35–41]. However, only one out of the 12 Pamphagidae species from Western and Central Anatolia analyzed here were X0/XX, the 11 remaining species showing a neo-XY sex chromosome system. As a whole, among the 55 species of Pamphagidae hitherto analyzed, 22 show neo-XY sex chromosome systems [11, 13–22, 35–41]. The high proportion (40%) of species with neo-sex chromosomes in Pamphagidae family is comparable with high portion (48,4%) of species with neo-sex chromosome in neotropical Melanoplinae species [8, 9, 42], thus pointing to a special role of sex chromosome-autosome fusion during the evolution of these groups of grasshoppers.

The high similarity in size and morphology of the neo-X chromosome between all species analyzed suggest the possibility that the ancestral autosomes, engaged in the centric fusion, were similar sized in both lineages. Furthermore, in early publications this fusion was considered monophyletic [19]. However, analysis of mitochondrial *COI* gene in Pamphagidae family showed that Trinchinae species and Nocarodeini species forms two different clades [43], which supports independent origin of neo-sex chromosomes in these two lineages. Thus, different types of neo-sex chromosomes in Trinchinae and Nocarodeini species could not be considered as different stages of one monophyletic process as it was suggested earlier. Taking in account these data, two independent evolutionary lineages of species with neo-XY sex chromosome systems were revealed in Pamphagidae grasshoppers. One lineage was observed in the studied species of *Glyphotmethis* (present paper), *Asiotmethis* [14, 19, 20, 22] and *Atrichotmethis* [18] genera, (belonging to the Trinchinae subfamily) and the other lineage was observed in all studied species of the Nocarodeini tribe [18, 19, 22] (belonging to the Pamphaginae subfamily). Impressive structural conservatism of neo-X chromosomes was observed in both evolutionary lineages. The submetacentric neo-X probably derived from centric fusion between an ancestral medium sized acrocentric X chromosome and large ancestral acrocentric autosome. The neo-Y chromosomes, by contrast, showed remarkable differences between species belonging to the two different evolutionary lineages. In *Glyphotmethis* and *Asiotmethis* genera, the size of the neo-Y chromosome was very similar to that of the XR arm of the neo-X chromosome (both being derived from the ancestral autosome pair), and consistently showed scarce C-banding. This indicates that these neo-Y chromosomes have scarcely evolved in respect to their autosomal ancestral condition, and we can consider that they are at the initial stage of heterochromatinization and differentiation (Fig. 1c, e, g). Additional evidence is provided by chiasma formation along the whole length of the neo-Y and the XR arm [5–7]. In Nocarodeini species, on the other hand, the size of the neo-Y chromosome was conspicuously smaller than that of the XR arm of the neo-X chromosome, and its proximal third is heterochromatic, a feature which is not observed in the corresponding regions of the XR arm (see Figs. 3, 4). The smaller size of the neo-Y suggests that it has lost part of the euchromatin of the original autosome. All these features, along with the fact that chiasma formation between the neo-Y and the XR arm is restricted to a single distal chiasma, indicates more advanced evolutionary stage of these neo-XY sex chromosome systems [5–7]. Initial stage of neo-sex chromosomes in Nocarodeini tribe was observed only in *Saxetania cultricollis* [18].

Trinchinae species with neo sex chromosomes inhabit Central Asia, China, and Bulgaria [14, 18–20, 22]. However, the highest biodiversity in Trinchinae species has been described in Western Asia [44], for which reason it is worth assuming that neo-XY formation is frequent in this region. The wider geographic distribution of some Trinchinae species with neo-sex chromosomes could be the result of intensive migration through arid planes of Eurasia.

Species from the Nocarodeini tribe are distributed in the Western Asian region and adjacent territories. Neo-sex chromosome systems have been found in all species of this tribe hitherto analyzed, including those previously karyotyped [2–4]. Intensive evolution of neo-sex chromosomes in Nocarodeini grasshoppers has led to variation including several types of neo-Y chromosomes and other derived sex chromosome systems. On the one hand, a neo-Y similar to the XR arm was described in *Saxetania cultricollis* [18], whereas *Paranothrotes opacus* shows a neo-X_1_X_1_X_2_X_2_/neo-X_1_X_2_Y sex chromosome system [22]. Remarkably, no species with the basal X0/XX sex chromosome system has yet been found in this tribe. We suggest that the X- autosome fusion took place in a common ancestor of the whole tribe and contributed to the divergence of this taxon. Evolution of the formed neo-sex chromosomes in Nocarodeini species is an ongoing and intensive process, as they appear to be at different evolutionary stages in different species.

### Cytogenetic features of Pamphagidae chromosomes

Karyotyping of Orthopteran families performed by White [1] led grasshoppers to be considered a classical example of karyotype stability. The present study in Pamphagidae grasshoppers has shown that about 60% of the species hitherto analyzed still conserve the ancestral X0/XX sex chromosome system, but the remaining 40% carry a derived neo-XY sex chromosome system. Leaving apart sex chromosome differences, the autosomes exhibit a high level of conservatism. The morphology and size of autosomes is rather similar among species. However, minor differences in autosome morphology were found. Small polymorphic short arms were observed in *Glyphotmethis* species. This feature was previously described in chromosomes of two other species also belonging to the Trinchinae subfamily, i.e. *Melanotmethis fuscipenis* [18] and *Eremopeza festiva* [22]. In contrast to the Trinchinae subfamily, in all karyotyped Pamphaginae species no apparent short arms were found. This difference might arise as a result of some events that took place after the divergence of these evolutionary linages.

Chromosome markers are necessary to study karyotype evolution. Unfortunately, the high variability in C-banding patterns does not make them useful markers. C-banding reveals regions enriched in repetitive DNA, but provides no information about the repeats contained in the C-blocks. Similar C-blocks may consist of different repeats. For instance, in a previous paper, we showed that, in the Gomphocerinae subfamily, most rDNA clusters revealed by FISH were located in interstitial C-bands [28]. In Pamphagidae grasshoppers, however, only 9 rDNA clusters co-localized with interstitial C-bands. Many clusters of rDNA were found on a pericentromeric C-positive region and also on C-negative regions (Fig. 4).

In groups of closely related species, such as the Gomphocerini tribe, the location of rDNA clusters can be a useful feature for identification of homeologous chromosomes [28]. However, high evolutionary mobility of the rDNA clusters has been described for many taxa [27, 45–49]. The mechanism of this high evolutionary mobility is unknown; it probably involves different chromosome rearrangements or insertion and amplification of rDNA units [50]. We observed high variation in the location of rDNA clusters in the Pamphagidae family. In all 12 species analyzed here, the only conserved location for rDNA clusters was found in the middle of the M_6_ autosome. This same location was previously described in *Asiotmethis turritus*, *Nocaracris cyanipes* and *Paranocaracris rubribes* [22]. In *G. adaliae* (a species with an XX/X0 sex chromosome system) we found an rDNA cluster in the middle of the M_7_, suggesting the possibility that the M_7_ chromosome in this species is probably homeologous of the M_6_ in all 15 species mentioned above. It would be interesting to analyze rDNA location in other X0/XX species to test this possibility.

The location of the rDNA clusters on large chromosomes was very variable, although they showed a tendency to be interstitially located. This variability is indicative of evolutionary changes that took place in these chromosomes but were not detected by the techniques used. Possible mechanisms explaining changes in rDNA cluster location could be paracentric inversions or the insertion of a DNA fragments containing rDNA into the chromosome, with subsequent rDNA amplification and elimination of the old rDNA cluster [50].

We should note that a different distribution of the rDNA clusters was revealed in *P. fieberi anatoliensis*. In contrast to all other studied species, the rDNA clusters were located in the pericentromeric region of six autosome pairs. This dramatic difference raises the question of its taxonomic status. Both *P. fieberi anatoliensis* and *P. fieberi tolunayi* are considered as subspecies of *P. fieberi* [51]. However, in contrast to *P. fieberi anatoliensis*, *P. fieberi tolunayi* showed the standard distribution of the rDNA clusters in the autosomes of other Nocarodeini species. The X chromosome of *P. fieberi anatoliensis* also differed from the X of other Nocarodeini species. In the *P. fieberi anatoliensis* XL arm, no rDNA cluster was found, whereas, with one exception (*P. turkmen*), all species of the Nocarodeini tribe carried an rDNA cluster in the XL arm. It is conceivable that an rDNA cluster was present in the ancestral acrocentric X chromosome, but it has been lost in *P. turkmen* and *P. fieberi anatoliensis* during evolution. Alternatively, the existence of other cryptic neo-XY lineages which have gone unnoticed with the present techniques is also possible. The different location of rDNA on the XL arm in the *Paranocaracris* and *Paranocarodes* genera might be explained by cryptic rearrangements or other mechanisms.

In the Trinchinae subfamily, analysis of rDNA distribution yielded little information on the evolution of the neo-sex chromosomes and, consequently, no hypothetic pattern of rDNA distribution in the ancestral X chromosome can be suggested.

The absence of an rDNA cluster in the XR arm of most species from both groups and in the neo-Y chromosomes may indicate that the ancestral autosome that entered the fusion and formed the XR arm and neo-Y chromosome did not carry the rDNA.

Apart from the telomeric repeats located in the termini of all chromosomes, we revealed telomeric repeats in the pericentromeric region of the neo-X in five out of 13 species carrying neo-sex chromosomes. The interstitial telomeric sequences (ITS) could be the result of chromosome fusions, unequal crossing-over or double strand break reparation involving telomerase (for review, see [52–54]). We suppose that ITS’s in the neo-sex chromosomes of these five species are the consequence of chromosome fusion. In case of fusion implying the loss of chromosome regions containing telomeric repeats in both chromosomes, no telomeric repeats would be included in the pericentric region of the resulting bi-armed chromosome. However, if the fusion would proceed yielding a dicentric chromosome, the possibility exists that the newly formed bi-armed chromosome would contain ITS’s in the pericentric region as remnants of the telomeric repeats placed beyond the centromere in the acrocentric chromosomes. It would thus be interesting to analyze whether the neo-X chromosome contains two pericentromeric regions and one active centromere in any of these species.

In Trinchinae species, the morphology and C-banding of neo-sex chromosomes indicates they are less evolved, and this might suggest that ITSs might be remnants of these centric fusion, which have been lost in some species. Due to their highly modified neo-Y chromosome, we consider that neo-sex chromosomes in Nocarodeini species are more evolved and therefore the ITSs in the pericentromeric region of the neo-X chromosome in most Nocarodeini species are absent, excepting *P. karabagi* and *P. fieberi*. We cannot distinguish whether they are remnants of telomeric sequences captured by the centric fusion yielding the neo-X chromosome or they are result of insertion and subsequent amplification of a DNA fragment containing telomeric repeats, or else the reparation of double strand breaks involving telomerase, however we think that later hypothesis is more probable. Also currently, *P. karabagi* and *P. fieberi* are classified into different genera. Therefore, we cannot rule out that these ITS’s may have resulted from independent events.

## Conclusion

Taken together, our present results show a high frequency of neo-XY sex chromosome systems in Pamphagidae grasshoppers inhabiting the Anatolian region, with at least two different lineages with independent neo-sex chromosome formation and evolutionary history. The first lineage is species of the Trinchinae subfamily characterized by evolutionary less advanced neo-sex chromosomes; the second lineage includes species of the Nocarodeini tribe and characterized by evolutionary advanced neo-sex chromosome. In the second lineage, the X-chromosome-autosome fusion may probably have taken place in the common ancestor of the Nocarodeini tribe. This family of grasshoppers thus shows one of the highest proportion of species carrying neo-XY sex chromosomes within Acridoidea grasshoppers. However, further research, including additional chromosome markers, is necessary to clarify the mechanisms for sex chromosome evolution in this group of grasshoppers.

## Abbreviations

FISH: Fluorescent in situ hybridization
ITS: Interstitial telomeric sequences
rDNA: ribosomal DNA
